# Compounds derived from bacteria enhance marine diatom growth

**DOI:** 10.1093/plphys/kiab139

**Published:** 2021-03-27

**Authors:** Ananya Mukherjee

**Affiliations:** Plant Science and Innovation, University of Nebraska Lincoln, Lincoln, Nebraska, USA

Diatoms are photosynthetic eukaryotes that are fundamental to aquatic food chains and fix one-fifth of global carbon. They store the fixed carbon in lipid form, making them desirable organisms for biofuel production (Wang and Seibert, 2017). However, the slow growth rate of diatoms in culture presents one of the main obstacles in the path of large-scale biofuel production (Khan et al., 2018; Sajjadi et al., 2018).

Co-cultivation studies have shown promise in enhancing diatom growth rates. Diatoms have close associations with other organisms, including bacteria, a fact that is illustrated by the abundance of bacterial genes that have been horizontally transferred into the genome of the model diatom *Phaeodactylum tricornutum* (Bowler et al., 2008). Previous studies have shown mutualistic relationships between bacteria and diatoms in which bacteria provide Vitamin B_12_ and siderophores (high-affinity iron chelators) in exchange for organic matter from diatoms (Hodson et al., 2007; Boyd and Ellwood, 2010). Better understanding of interactions between marine bacteria and diatoms can hold the key to improving diatom growth and lipid production.

In this issue of *Plant Physiology*, Sittmann et al. (2021) investigated how members of the *Bacillus cereus* group (a small group of closely-related bacteria species) can increase diatom cell count and lipid production. The authors co-cultured *P. tricornutum* with different bacteria from the *B. cereus* group to investigate the effect of these bacteria on diatom growth. The *B. cereus* group of bacteria are Gram positive, facultative anaerobes found everywhere from soil to water, animals, plants, and marine environments. They are known for their capacity to survive and adapt, form spores, and thus disperse widely (Guinebretière et al., 2008).

**Figure 1:**
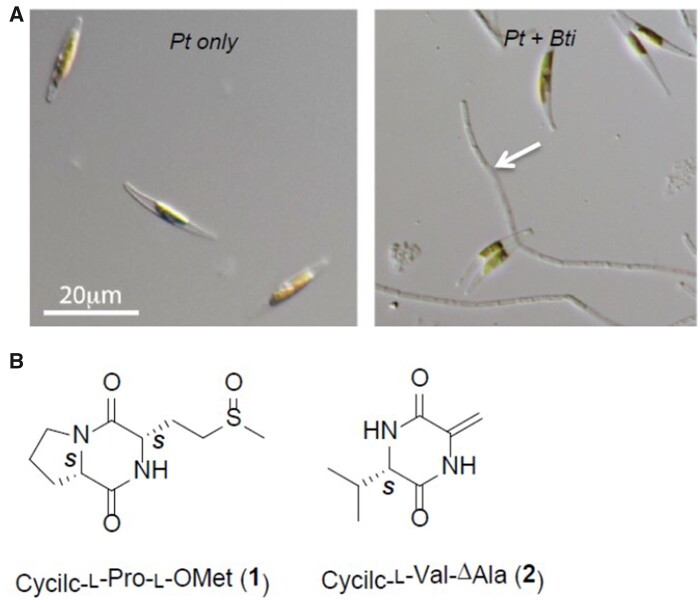
*Bacillus thuringenesis* (Bti) spores enhance the growth of *P. tricornutum* (Pt). A, Comparison of Pt cells and Pt with Bti under the microscope. The arrow shows spores of Bti that increase growth rate. B, Structure of the DKPs ((**1**) cyclic-L-Pro-L-*O*Met and (**2**) cyclic-L-Val-Δala) derived from sporulated Bti that play a role in increasing diatom biomass. Figure has been adapted from Sittmann et al. (2021).

The authors found that one type of bacteria tested, *Bacillus thuringiensis*, specifically enhanced diatom growth by two to three times, whereas other species from this group had little to no effect on diatom growth. Microscopic examination revealed the bacteria in the co-culture sporulated, and the timing of sporulation coincided with the increase in growth rate (Figure 1A). Sporulation is a state of dormancy that can result from nutrient stress, and the authors conclude the poor nutrient support for the bacteria provided by the diatom growth medium causes them to sporulate.

Sporulation is a multistep process that includes an asymmetric cell division that gives rise to a larger mother cell and an endospore. In the final stage of sporulation, the mother cell is lysed by programmed cell death. The authors showed increased diatom growth is caused by mother cell lysis, rather than simply the presence of spores, and lysis of the mother cell released small, heat-labile growth stimulating factors into the growth medium, thereby enhancing diatom growth.

Using reverse-phase solid-phase extraction, the growth-promoting compounds in the lysate were extracted. Comprehensive analysis of UV, mass spectrometry, and   Nuclear Magnetic Resonance (NMR) spectroscopy data indicated the compounds (**1** and **2**; Figure 1B) were diketopiperazines (DKPs), which are natural peptide derivatives recognized by a variety of receptors. DKPs are involved in quorum sensing, ion transport, and several other biological activities (Harizani et al., 2020). Further NMR analysis revealed compound 1 is cyclic-L-Pro-L-*O*Met, which is commonly found in marine sediment bacteria, and compound 2 is cyclic-L-Val-ΔAla originally identified from *Psuedomonas aeruginosa*. Cyclic-L-Val-ΔAla has been recognized as an antagonizer of quorum-sensing systems and thus affects signaling between bacterial species.

To test for the effect of bacterial lysate on diatom lipid accumulation, Gas chromatography–mass spectrometry (GC-MS) was used to analyze the yield and lipid composition of diatoms grown in co-cultures. After Day 7, an increase in the biodiesel components palmitoleic acid (C16:1), oleic acid (C18:1), and linoleic acid (C18:2) was observed along with some beneficial dietary lipids. Levels of unsaturated fatty acids increased but not saturated fatty acids. This effect has been shown previously in diatoms reacting to cold temperature or reduced nitrogen levels (Jiang and Gao, 2004). Diatoms grown in co-culture also produced higher levels of neutral lipids, such as triglycerides, compared to diatoms grown alone.

DKPs are involved in communication between organisms: some affect quorum sensing in bacteria, some can mimic plant growth regulators, and the only known DKP from diatoms acts as a pheromone involved in mating. This study demonstrates an additional role for naturally occurring DKPs, although many questions remain, including why they are produced in sporulating *B. thuringiensis* and how they stimulate growth and lipid accumulation in diatoms. This study also demonstrates that a *P. tricornutum–B. thuringiensis* co-culture can be productive for large-scale biofuel production and provides an alternative to genetic engineering lipid accumulation pathways in diatoms.
